# 

**Published:** 1856-09

**Authors:** 


### CONTENTS.

ORIGINAL COMMUNICATIONS— PAGE.

Report of the Committee on Practical Medicine. By Sam'l Thompson, M.D., of Albion, Edwards Co., Ill. .......... 391

Annual Address of the President of the Illinois State Medical Society, read at the Meeting in Vandalia, June 4, '56. By N. S. Davis, M.D. 406

Amputation at the Pubes for Cancer of the Penis. By N. Sherman, M.D. 422

SELECTIONS, .......... 425

BOOK NOTICES, .......... 432

EDITORIAL, .......... 433

